# Biliary Leak in Post-Liver-Transplant Patients: Is There Any Place for Metal Stent?

**DOI:** 10.1155/2012/684172

**Published:** 2012-05-01

**Authors:** Fernanda P. Martins, Melissa Phillips, Monica R. Gaidhane, Timothy Schmitt, Michel Kahaleh

**Affiliations:** ^1^Endoscopy Unit, Hospital Israelita Albert Einstein, 05653120 São Paulo, SP, Brazil; ^2^Department of Surgery, University of Tennessee Medical Center, Knoxville, TN 37920, USA; ^3^Division of Gastroenterology and Hepatology, Weill Cornell Medical Center, New York, NY 10021, USA; ^4^Division of Gastroenterology & Hepatology, Department of Surgery, University of Virginia Health System, Charlottesville, VA 22908, USA

## Abstract

*Objectives*. Endoscopic management of bile leak after orthotopic liver transplant (OLT) is widely accepted. Preliminary studies demonstrated encouraging results for covered self-expandable metal stents (CSEMS) in complex bile leaks. *Methods*. Thirty-one patients with post-OLT bile leaks underwent endoscopic temporary placement of CSEMS (3 partially CSEMS , 18 fully CSEMS with fins and 10 fully CSEMS with flare ends) between December 2003 and December 2010. Long-term clinical success and safety were evaluated. *Results*. Median stent indwelling and follow-up were 89 and 1,353 days for PCSEMS, 102 and 849 for FCSEMS with fins and 98 and 203 for FCSEMS with flare ends. Clinical success was achieved in 100%, 77.8%, and 70%, respectively. Postplacement complications: cholangitis (1) and proximal migration (1), both in the FCSEMS with fins. Postremoval complications were biliary strictures requiring drainage: PCSEMS (1), FCSEMS with fins (6) and with flare ends (1). There was no significant differences in the FCSEMS groups regarding clinical success, age, gender, leak location, previous treatment, stent indwelling, and complications. *Conclusion*. Temporary placement of CSEMS is effective to treat post-OLT biliary leaks. However, a high number of post removal biliary strictures occurred especially in the FCSEMS with fins. CSEMS cannot be recommended in this patient population.

## 1. Introduction

Biliary complications are frequent after orthotopic liver transplantation (OLT), affecting 5 to 15% of patients after deceased OLT and 28 to 32% after right-lobe living donor OLT [1–4]. Post-OLT bile leaks are reported in 10 to 15% of patients and are usually an early complication and represent a high morbidity condition for the patient [5].

Endoscopic treatment is well recognized as first-line therapy in the management of post-OLT biliary leaks or stricture [1, 4, 6, 7]. Endoscopic retrograde cholangiography (ERC) with biliary sphincterotomy and/or transpapillary plastic stent placement is typically offered as standard treatment [8–10]. Successful endoscopic therapy for postsurgical bile leaks has been reported in 88 to 97% of cases and up to 83.9% in those secondary to OLT [11]. 

Covered self-expandable metallic stents (CSEMSs) have been increasingly used to treat benign biliary conditions and have been shown promising results for both biliary strictures and leakages [7, 12–14]. Their larger diameter, long-term patency, and proven removability have turned them into an appealing option to assess refractory and/or complex bile leaks [7, 13–15]. 

Although few studies have already demonstrated encouraging results for CSEMS use in the treatment of postsurgical bile leaks [13, 14], their long-term safety and efficacy have not been established for this particular group of patients when compared to plastic stents. Indeed in order for CSEMS to replace plastic stents in this indication, not only they need to demonstrate efficacy but also long-term safety.

Therefore, the primary aim of this study was to review our experience with temporary placement of three different types of CSEMS in the treatment of post-OLT biliary leaks.

## 2. Materials and Methods

We retrospectively reviewed a prospectively established database to assess post-OLT bile leaks treated with CSEMS between December 2003 and December 2010.

Inclusion criteria were the same as previously defined and included patients with complex or high-grade leaks defined as those who failed endoscopic plastic stent therapy or with severe comorbidities that prevented multiple procedures who were referred to temporary placement of CSEMS [13]. One patient included in the PCSEMS group has been previously reported [13]. Data was captured prospectively as each type of stent came on the market in chronological order (first partially covered, then fully covered with fins, after that fully covered with flared ends). Stent diameter was chosen depending on diameter of the ducts to be drained.

Our endpoints were long-term clinical success (resolution of bile duct leak) and safety.

Our Institutional Review Board approved the study, and written informed consent was obtained from all patients prior to ERC.

### 2.1. Techniques of CSEMS Placement

**Figure 1:**
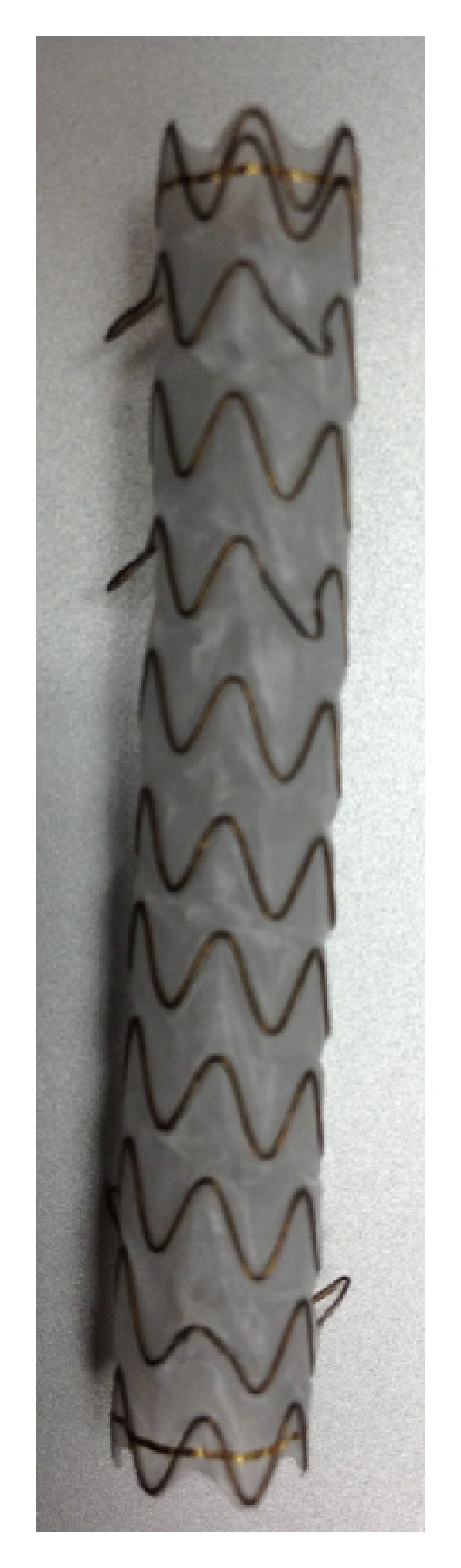
Fully covered SEMS with antimigratory fins.

**Figure 2:**
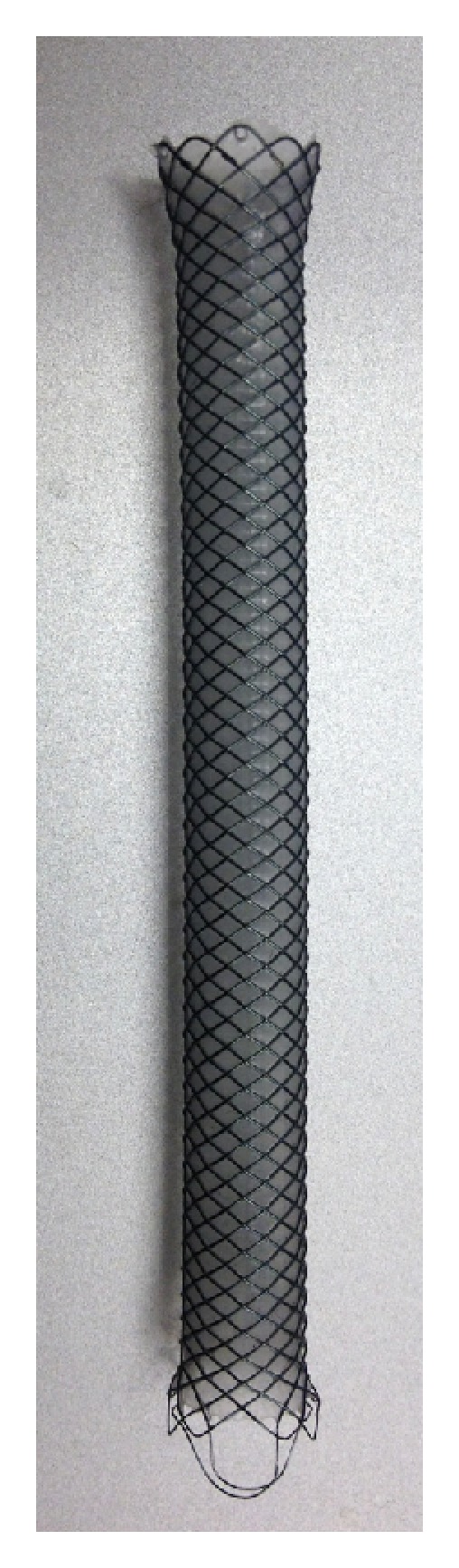
Fully covered SEMS with flare end and retrieval loop.

All procedures were either performed or supervised by dedicated biliary endoscopists performing at least 500 ERC yearly. ERC was performed under general anesthesia with patients in the supine position. Side-viewing endoscopes (TJF-140, TJF-160 and TJF-160VF, Olympus America, Center Valley, PA, USA) were used for all procedures. Three different types of CSEMS were placed: partially CSEMS (PCSEMS) (Wallstent, Boston Scientific Corp, Natick, MA, USA), fully CSEMS (FCSEMS) with fins (Viabil, Conmed, Utica, NY, USA) (Figure 1), and FCSEMS with flared ends (WallFlex, Boston Scientific Corp; Figure 2).

**Figure 3:**
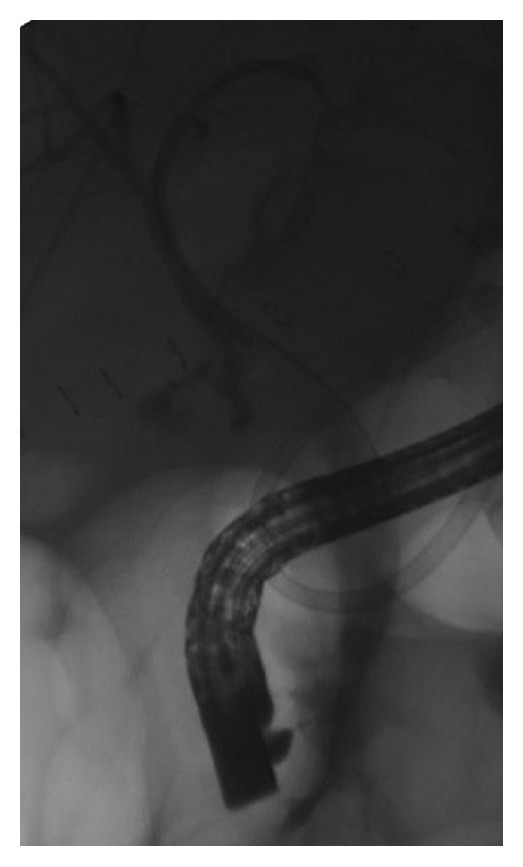
Anastomotic leak s/p liver transplant.

**Figure 4:**
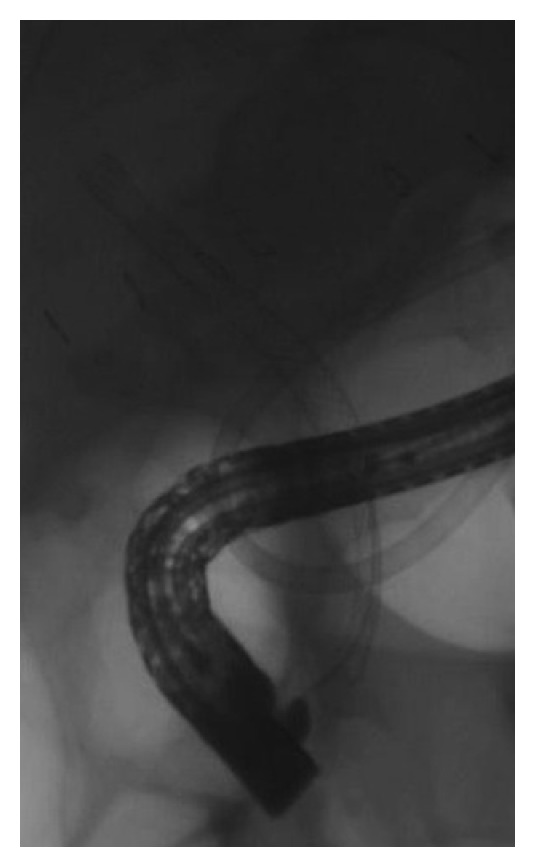
Fully covered SEMS deployed.

After selective biliary cannulation, biliary sphincterotomy was performed, and a retrieval balloon was used to perform an occlusion cholangiogram and locate the bile leak (Figure 3). Over the guidewire placed across the leak, the CSEMS was deployed under fluoroscopic control sealing the leak (Figure 4). The stent was placed, crossing the papilla for at least 1 cm after deployment.

### 2.2. Definition of Events

Successful CSEMS placement was defined as deployment of the CSEMS across the leak with resolution of the leak fluoroscopically. Proximal migration of the FCSEMS was defined as any migration of the CSEMS into the bile duct. Distal migration was defined as migration of the stent into the duodenum from the transpapillary position. Spontaneous CSEMS migration with resolution of the leak was recorded but was not considered a complication. Post-stent-removal biliary strictures were defined as a narrowing demonstrated on imaging associated with elevated liver function tests.

### 2.3. Followup after CSEMS Placement

All patients were seen in our liver transplant clinic and digestive health center with consultation of nephrology, pathology, or infectious diseases whenever indicated. Short-term followup was obtained by a clinic visit, with cross-sectional imaging one month after the FCSEMS was removed. Laboratory values, including complete blood count and hepatic function panels, were also closely followed. Long-term followup was obtained either by clinic visit or telephone interview.

### 2.4. Technique of CSEMS Removal

After leakage resolution, CSEMS was removed by using the rat tooth and/or snare technique as previously described [16]. In cases where the CSEMS had foreshortened or migrated within the bile duct, balloon dilation was used to disimpact the CSEMS with subsequent rat tooth removal [17]. This was particularly important when dealing with the partially CSEMS due to the development of tissue overgrowth at the proximal portion, imbedding the CSEMS or when removing the FCSEMS with fins, which anchor the CSEMS within the bile duct. The FCSEMS has a loop at its distal end, which permits extraction using a rat tooth, and, unless the loop was imbedded within the ampullary tissue, this technique was used preferentially.

Choledochoscopy was performed in patients whenever a lesion or a stricture was suspected on fluoroscopy following CSEMS removal and used the single operator system (SpyGlass, Boston Scientific).

### 2.5. Data Collection and Statistical Analysis

Data was collected from electronic medical records and our dedicated procedure database. Data was captured prospectively on all patients and analyzed retrospectively. Clinical response to CSEMS placement and procedure-related morbidity and mortality rates were analyzed.

SAS 9.2 (SAS Institute Inc. 2008, Cary, NC, USA) was used for statistical analysis. Descriptive data were expressed in means, medians, and standard deviations (SDs). Fischer's exact tests were conducted to observe any significant differences between stent groups, with the statistical significance set at *P* < 0.05.

## 3. Results

During the study period a total of 451 transplants were performed. A choledochocholedochostomy was performed in 428 patients (95%) while 23 patients received a roux anastomosis (5%). Posttransplant leak was observed in 54 (12%) patients and ischemic cholangitis in 23 (5%) patients.

Thirty-one patients underwent CSEMS placement for bile leak treatment after deceased liver transplant, three in the PCSEMS group, eighteen in the FCSEMS with fins group, and ten in the FCSEMS with flare ends group. Patients' demographic characteristics are summarized in Table 1. There was no evidence of significant differences among them regarding age, gender, leak site, time interval between OLT, and procedure to CSEMS deployment and previous treatment with plastic stent. All patients had a single duct-to-duct anastomosis, and there was no reference to complex arterial reconstruction among patients treated in this series.

### 3.1. Partially CSEMS Group

Partially CSEMSs (80 mm length) were temporary placed in three patients for a median time of 89 days (range 55 to 110). After a median followup of 1,353 days (range 1,348 to 2,208), clinical success was achieved in all patients. There was one case of spontaneous migration, even though the patient had the leak resolved. Another patient developed a late-onset hilar stricture, refractory to both endoscopic plastic stenting therapy and underwent hepaticojejunostomy. Choledochoscopy was performed for that patient prior to surgery confirming tissue overgrowth at the hilum.

### 3.2. Fully CSEMS with Fins Group

**Figure 5:**
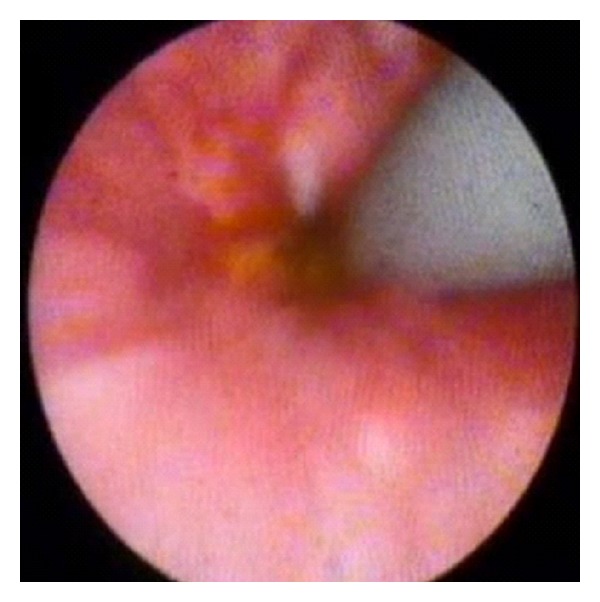
Ulceration seen during choledochoscopy after fully covered SEMS removal.

**Figure 6:**
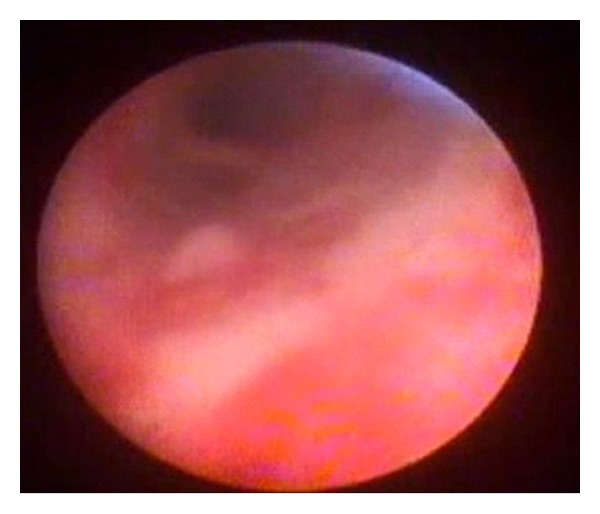
Biliary stricture seen during choledochoscopy after fully covered SEMS placement.

In this group, 18 patients underwent temporary placement of the FCSEMS with fins (80 and 100 mm length) for a median of 102 days (range 35 to 427) and were followed after removal for a median of 849 days (range 323 to 1,111). Long-term leakage control was achieved in 14 patients, 77.8% in an intention to treat analysis. One patient died from unrelated cause, and 2 underwent liver retransplantation due to hepatic artery thrombosis; by excluding them from the long-term analysis, the clinical success rate would be 93.3% (14/15). One patient presented cholangitis after stent deployment and was treated with repeated ERC and plastic stent placement. Post-stent-removal complications included six clinically significant hilar biliary strictures that required biliary drainage (6 plastic with plastic stents, 2 with CSEMS, and 1 underwent a surgical hepaticojejunostomy). Choledochoscopy was performed in five patients and demonstrated ulcerations (Figure 5) in 4 patients (80%) that were managed conservatively and one (20%) hilar stricture (Figure 6) that was treated with plastic stenting.

### 3.3. Fully CSEMS with Flare Ends Group

Ten patients received a FCSEMS with flare ends (80 mm length). The CSEMS was kept in place for a median time of 98 days (range 96 to 139), and 3 patients still have the stent in situ. Median followup after stent removal was 203 days (range 95 to 305), and so far 70% (7/10) of patients presented clinical resolution of biliary leakage. One patient presented stent migration and spontaneous passage, with leak resolution. One patient (10%) developed a hilar stricture after CSEMS removal and received plastic stenting. Four patients underwent choledochoscopy during stent removal, which revealed inflammation in two patients (20%) and ulceration in one (10%), but no hyperplasia.

### 3.4. Statistical Analysis

Fischer's exact test was conducted to analyze for differences in clinical success rates (leak resolution) among the FCSEMS groups. The PCSEMS group was excluded due to a small number of patients. There was no evidence of significant differences in the 2 stent groups with regards to clinical success, age, gender, leak location (anastomotic or non-anastomotic), previous treatment with plastic stent, stent indwelling, postplacement, and post-removal complications (all *P* > 0.05). Followup was significantly longer in the FCSEMS with fins group (*P* < 0.01).

## 4. Discussion

Biliary leaks occur in 10 to 15% of patients after OLT and usually present with clinical symptoms earlier than strictures in the postoperative course [11]. They are typically classified into anastomotic or nonanastomotic.

Endoscopy stands as a first-line treatment for post-OLT biliary leaks [11, 18]. However, many anastomotic leaks may require surgical repair [11] and, therefore, have been defined as a complex leak [13, 14]. 

The main principle of endoscopic therapy for biliary leakage is to reduce transpapillary pressure gradient via transpapillary stenting with or without biliary sphincterotomy; this is conventionally performed using plastic stents [8, 9, 11].

With the large diameter provided by metal stent and the ability to remove covered metal stents, the use of CSEMS in bile leaks was the logical next step.

This was initially described by Baron and Poterucha [15], when they reported 3 cases of complex bile leaks successfully treated with CSEMS.

Promising results of CSEMS have also been further reported by our team with long-term leak control obtained in 87% (14/16) of patients with post-surgical bile leaks [13]; however, long-term results were not available yet.

CSEMSs have mainly been used as a rescue therapy for patients who failed standard endoscopic therapy with plastic stenting. However, more recently has been used as a first-line measure in patients with complex and high-grade leaks [19, 20].

The rationale to deploy a SEMS through a leak is to grant the larger diameter possible allowing the patient to have the faster recovery time, with fewer sessions, and prevent further complications associated with the bile leak [21].

The median indwelling of CSEMS in this study was 189, 102, and 98 days, respectively, for the PCSEMS, the FCSEMS with fins, and the FCSEMS with flare ends, respectively. The use of CSEMS for the treatment of bile leak has the theoretical advantage of decreasing the number of procedures needed to control the leakage in patients with complex or high-grade fistulas when compared to plastic stents. It is presumed that the initial increased cost associated with the use of CSEMS will be compensated by the reduction of sessions required as well as the days of hospitalization. This last point, however, remains to be proven.

 All patients in the partially CSEMS group had previously failed plastic stenting (i.e., persistent leak after plastic stent placement), and, after placement of the metal stent, leak control was achieved in all of them (Tables 1 and 2).

Wallstent was the first PCSEMS commercially available in the US market but led to mucosal hyperplasia at its uncovered portions and migration [12, 13]. Proximal migration is especially problematic since it can be associated with hyperplasia and secondary stricture after stent removal. Distal migration can lead to treatment failure.

Isayama et al. studied both radial (RF) and axial forces (AF) of Wallstent and Viabil [21]. The results demonstrated Wallstent to have high AF, possibly related to biliary wall damage, kinking, and sludge formation and migration [21]. In the present study, distal migration was noted in one patient (33.3%) from PCSEMS and one (10%) from FCSEMS with flare ends group. This migration rate has been reported in other studies [20, 22] and might be related to the respective force of the 2 CSEMSs, which are similar (Unpublished data from Isayama).

The FCSEMS with anchoring fins positioned at opposite ends was designed to prevent migration [14]. It was found to have a very high RF in Isayama et al. study, what might cause an excessive high pressure to the biliary wall, resulting in increased tissue injury and eventual stricture [21]. In this study, tissue injury was found in 4/5 (80%) patients when choledochoscopy was performed in this group and an increased number of post-stent-removal strictures (35%) that could be a consequence of biliary compression and ischemia. Even though migration rate was lower (5.6%), the increased number of postremoval stenosis is disencouraging.

A discussion is raised if the incidence of postremoval strictures was related to the high radial force or to the presence of fins that could stimulate tissue reaction and scarring.

Finally, FCSEMS with flare ends was recently released as fully covered SEMS, coated with premalume. It has a retrieval loop and flare ends to prevent migration. Although the leakage controlled has been encouraging in this study, we are still dealing with spontaneous distal migration (10%) and postremoval stricture (10%).

Interestingly there no statistically significant difference in either group in term of final outcome; this might be related to a type 2 error, which can be overcome by increasing the number of patients in each sample; however the complications' rate associated with all three CSEMS does not justify such a study.

Clearly, the ideal CSEMS for biliary leak is not available yet. It probably needs to be fully covered with an inert and resistant coating and has no fins, which seem to be associated to significant tissue reaction. Further CSEMS soon invading the market might offer those characteristics and need to be carefully evaluated.

In conclusion, temporary placement of CSEMS was effective to treat post-OLT biliary leaks. However, postremoval biliary stricture requiring further endoscopic treatment was seen especially with the FCSEMS with fins group. At the present time, CSEMS cannot be recommended in this patient population until major design changes have been made.

## Figures and Tables

**Table 1:** Patients demographics.

Type of stent	PCSEMS	FCSEMS with fins	FCSEMS with flare ends
N	3	18	10

Gender			
Male	3	15	7

Age			
(mean ± SD)	50 ± 8.7	53.9 ± 5.0	56 ± 6.7
Median (range)	40–56	42–60	48–69

Underlying liver disease			
HCV		6	4
Ethanol		3	1
Cryptogenic	2	3	2
HCV + ethanol		2	2
HBV		1	
NASH	1	1	1
Other		2	

Post-OLT leak location			
Anastomotic	1 (33.3%)	14 (77.8%)	7 (70%)
Nonanastomotic	2 (66.7%)	4 (22.2%)	3 (30%)

Time OLT to procedure (days)			
Mean (SD)	36 (±8)	53.2 (±85.8)	22.9 (±16.4)
Median (range)	33 (29–45)	20 (6–328)	19 (5–63)

Previous plastic stenting			
Yes	100%	3 (16.7%)	2 (20%)
T-tube	0	0	1 (10%)

OLT: orthotopic liver transplant, SD: standard deviation, HCV: hepatitis C virus; HBC: hepatitis B virus; others (hemochromatosis, alpha-1-antitrypsin). PCSEMS: partially covered self-expanding metal stent, FCSEMS: fully covered self-expanding metal stent.

**Table 2:** Long-term evaluation of SEMS for the treatment of biliary leaks.

	PCSEMS	FCSEMS with fins	FCSEMS with flare ends
N	3	18	10

Stent in place (days)			
Mean (SD)	85 (±28)	152.3 (±117.6)	106 (±19.7)
Median (range)	89 (55–110)	102 (35–427)	98 (96–139)

Stent diameter			
8 mm	0	2	5
10 mm	3	16	5

Stent status			
Stents removed	2 (66.7%)	13 (87%)	7 (70%)

Followup after removal (days)			
Mean (SD)	1636.3 (±495.1)	797.9 (±261.9)	189.4 (±83.5)
Median (range)	1353 (1348–2208)	849 (323–1111)	203 (95–305)

Long-term success			
Intention to treat	3/3 (100%)	14/18 (77.8%)	7/10 (70%)

Postplacement complications			
Cholangitis	0	1/18 (5.6%)	0
Proximal migration	0	1/18 (5.6%)	0

Postremoval complications			
Biliary stricture	1/3 (33.3%)	6/18 (35%)	1/10 (10%)

SD: standard deviation.
